# A cross-sectional study on preferred employment settings of final-year nursing students in Israel

**DOI:** 10.1186/s12960-020-00496-6

**Published:** 2020-07-31

**Authors:** Yael Sela, Keren Grinberg, Yair Shapiro, Rachel Nissanholtz-Gannot

**Affiliations:** 1 Nursing Department, Faculty of Social and Community Sciences, Ruppin Academic Center, Emeq-Hefer, Israel; 2 Department of Health Management, School of Health Sciences, Ariel University, Ariel, Israel; 3 Smokler Center for Health Policy Research, Myers-JDC-Brookdale Institute, Jerusalem, Israel

**Keywords:** Community care, Hospital, Nursing students, Nursing education

## Abstract

**Background:**

Despite the growing demand for community nurses, their number remains relatively low. We examined perceptions of final-year nursing students regarding their preferred work setting after graduation and the factors affecting their choice.

**Methods:**

A cross-sectional survey using a structured questionnaire was developed specifically for this study. The questionnaire was distributed among fourth-year students from all nursing training frameworks across Israel.

**Results:**

Of 281 respondents (76.6% women, average age, 29.3 years), most (80.9%) preferred working in hospitals, while 5% preferred community settings; 14% were undecided. Students’ knowledge on hospital nurses’ tasks was greater compared to their knowledge on community nurses’ tasks. Moreover, hospital nurses’ tasks were perceived as more important than those of community nurses. The contribution of clinical placement in hospital nursing was perceived as significantly more meaningful than the contribution of clinical placement in community nursing. The vast majority of students (94.3%) stated that they would prefer to undergo a hospital nursing internship. A significant correlation was noted between students’ clinical placement, the exposure to community nursing roles, and the perception of the community nurse’s role: clinical placements that were perceived as a positive experience led to a more positive perception of community nurses’ roles.

**Conclusions:**

Nursing students’ perception of community nursing is based upon limited information which does not reflect community nurses’ actual role and work.

## Background

Recent decades have seen a growing need for provision of healthcare services within the community due to the growth in aging populations, increased rate of chronic diseases and co-morbidities, reduced length of hospital stays, and the shift of care from hospitals to community and home care [1, 2]. Although these changes require more nurses for work in community settings, a significant shortage in community nurses (also called public health nurses) has been reported in many countries [3–7]. The reasons cited for this shortage include challenges in attracting nurses to this field due to invisibility of community nursing in media and marketing campaigns [8], the misperception that community healthcare provides less opportunities for professional development and that it has a lower status than inpatient nursing [9], inconsistencies in educational approaches to community nursing, lack of clinical training sites [10], and a shortage of nursing faculty adequately prepared to teach community nursing [8]. Additionally, many community nurses leave this field due to job discontent, unit staffing inadequacies [11], and lower salaries paid to nurses by governmental agencies compared to hospitals [9]. The imminent retirement of older nurses is also expected to contribute to the shortage of community nurses [9].

According to the World Health Organization, nurses that provide healthcare services in the community have major roles in promoting health, preventing injury and disease, mitigating disability, and providing and managing care and follow-up in multiple settings [12]. The role and responsibilities of community nurses vary among countries. About 70% of the nursing workforce in Israel work in hospitals, 20% work in community nursing, and approximately 5% hold administrative positions, or work in research, nursing training institutions or universities [13, 14]. Among the nurses who work in the community, most are employed in primary care clinics belonging to the four health plans that provide public health care to all Israeli citizens through the National Health Insurance Law [15]. Other community nurses are employed by a Ministry of Health-funded network of preventive mother and child health centers [13, 14]. The main areas of nurses’ activities in health plans include routine and chronic care, quality monitoring and improvement, specialized care (e.g., diabetes and geriatric care), home care, and health promotion. In addition, community nurses are increasingly involved in inter-professional education of primary care teams, are being appointed to general management positions (e.g., regional and program directors), and are given lead roles in hospital-community transitions and health promotion [1].

Israel has several nursing training programs: academic (4-year program) and non-academic (2.5–3-year program). These programs are based on the Nursing Administration’s core curriculum, two thirds of which are classroom (theoretical) courses, and about one third are practical (clinical) studies. The curriculum comprises a basic science division, a nursing science division, and a clinical studies division comprising classroom courses, clinical placement, and an internship. In addition to teaching this core curriculum, each nursing program director may add various additional contents. To register as a nurse (RN) at the Ministry of Health, the student must complete the core program and successfully pass the governmental exam in nursing. A nurse who graduates from the core program can work in almost all departments, but her authorities are limited to specific activities. RNs with an academic degree in nursing can take additional courses in specific clinical fields and must pass an exam in order to expand their authorities and responsibilities [16].

During the course of their studies, students undergo critical socialization processes during which they formulate worldviews on the nurse’s role. These processes assist in understanding and defining the student’s expectations of the nurse’s role. This is not just a matter of acquiring technical skills, but also of understanding the values, norms, and fundamentals of the profession [17, 18]. These processes greatly influence the choice of work setting upon graduation, and sometimes, the decision to remain in the nursing profession.

Examination of the process by which nursing students formulate their views and perceptions on the nursing profession, and the factors influencing their choice of work setting after graduation from nursing training programs, is expected to assist in policy making and strategic planning at all levels of the healthcare system for directing more nurses to choose community nursing. In this study, we evaluated the perceptions and attitudes of final-year nursing students concerning their preferred work setting after graduation, and the factors that contribute to their choices.

## Materials and methods

This cross-sectional study was approved by Ariel University’s Ethics Committee, and informed consent was obtained from all participants in the study. The participants’ responses were confidential and were not linked to their identity.

### Population and sample

The survey was conducted among fourth (final) year nursing students from all nursing training frameworks across Israel.

During the study period, the average number of new nursing licenses was 1142 licenses per year. Given the size of the population in Israel (~ 8 million at the time of the study), the assumption of a response rate of 70%, and considering a confidence interval of 0.95, a sample size of 350–400 participants was calculated.

### Data collection tool

The structured questionnaire used in this study was based on a literature review and on relevant issues and themes that were raised in a previous qualitative study that evaluated the perceptions of senior medical managers, nursing managers, and policymakers on career choices of nursing students after graduation [19]. The questionnaire was reviewed by several experts from the fields of nursing and health system management and was revised according to their comments.

To evaluate the questionnaire’s reliability, it was distributed among 30 nursing students. Questions that received a Cronbach’s alpha coefficient of less than 0.6 were excluded from the questionnaire. The final questionnaire covered the following topics: demographics, students’ perceptions of nurses’ roles in the hospital, students’ perceptions of nurses’ roles in the community, students’ considerations when choosing their work setting after graduation, and nursing school curriculum and training.

### Data collection

The structured questionnaire was distributed to nursing students between September 2015 and January 2016. The students completed the questionnaire during class. Before distributing the questionnaire, the researcher emphasized to the students that participation is voluntary and anonymous.

### Data analysis

The collected data were analyzed with descriptive and inferential statistics. *T* test was used to examine differences between two categories. Chi-squared test was used to compare categorical variables. Spearman coefficients were computed to measure correlations between ordinal variables. Multiple linear regression with stepwise selection (*α* = 0.15) was utilized to determine the most contributing factors to predict the mean relative score difference between students who prefer working in community nursing and those who prefer the hospital setting. The regression models included variables that were significantly associated with the dependent variable in the univariate analysis (*p* < 0.05). Interactions between the independent variables were also tested within the regression model. All statistical comparisons were two-sided with significance defined as *p* < 0.05.

## Results

Of the 345 nursing students that were approached by the researcher, 281 students (81.5%) provided complete questionnaires and were included in the analysis. The respondents’ demographics are shown in Table 1. Most of the students (88.3%) were in undergraduate nursing programs (Bachelor of Science in Nursing that includes Registered Nurse certification), were women (76.6%), and were born in Israel (75.9%). The participants’ age ranged from 20 to 55 years (average, 29.3 years, standard deviation, 5.9). About two thirds of the participants (66.5%) were Jewish and about a quarter (25.6%) were Arab Muslims. Over half of the participants were single (53.2%), and 45% were married.

**Table 1:** Respondents’ demographics

Variable	Respondents ***N*** = 281 ***n*** (%)
**Gender**	
Females	215 (76.6%)
Males	66 (23.4%)
**Country of birth**	
Israel	211 (75.1%)
Russia and former Soviet Union States	54 (19.2%)
United States of America	3 (1.1%)
Italy	2 (0.7%)
Not specified	11 (3.9%)
**Ethnicity**	
Jewish	187 (66.5%)
Muslim	72 (25.6%)
Other	22 (7.8%)
**Marital status**	
Single	150 (53.2%)
Married	127 (45.0%)
Divorced	5 (1.8%)
**Type of nursing training program**	
Undergraduate	225 (88.3%)
Graduate entry	45 (16.7%)

### Considerations for choosing a work setting after graduation

Among the students who participated in the study, 59.7% (166/278) claimed that at the beginning of their nursing studies they would have preferred to work in a hospital after graduation. This rate rose to 80.9% (225/278) in the fourth year of nursing studies. On the other hand, 4.3% (12/278) of the students claimed that at the beginning of their studies they would have preferred community care, while 5.0% (14/278) preferred community care in the fourth year. Seventeen percent of respondents (47/278) claimed to have been undecided in their first year, and 14% (39/278) were still undecided regarding their preferred work setting after graduation. The vast majority of respondents (94.3%, 249/263) stated that they would like to undergo their nursing internship in a hospital.

Respondents, who stated they would like to work in a hospital after graduation, were asked to specify their preferred department. Of 153 respondents who responded to this question, 23.5% (36/153) preferred working in surgical departments, 20.2% (31/153)—pediatric departments, 19.6% (30/153)—emergency medicine, 16.3% (25/153)—internal medicine, and 15.6% (24/153)—gynecology and obstetrics. Only 3.3% of the respondents (5/153) replied that they would like to work in psychiatric departments, while 1.3% (2/153) replied that they would like to work in geriatric departments. Similar rates were noted when the respondents were asked about their preferred department during internship.

All of the respondents whose clinical placement during their studies included working in surgical departments replied that they would prefer working in a hospital after completing their studies. Similarly, most of the respondents whose clinical placement included working in internal, emergency, pediatric, geriatric, and psychiatric departments stated that they would prefer working in the hospital after graduation. Of the 14 students who had worked in community care, 8 (57.1%) stated that they would prefer working in this field (Table 2).

**Table 2:** Influence of clinical experience during nursing studies on preferred work setting for nursing internship

Preferred work setting for nursing internship	Students with clinical experience in the department during their studies	Students who indicated the hospital as their preferred work setting after graduation	Students who indicated the community as their preferred work setting after graduation	Undecided
**Hospital**				
Surgery (including operating rooms)	51	51	0	0
Internal medicine, oncology, cardiology	48	40	1	7
Emergency medicine, intensive care, trauma	60	58	1	1
Midwifery and gynecology	39	35	3	1
Pediatrics (including pediatric hematology and oncology, pediatric emergency medicine)	34	33	0	1
Geriatrics	1	1	0	0
Psychiatry	2	2	0	0
**Community**				
Health plans, public health clinics	4	4	8	2

When asked to rate their considerations when choosing their preferred work setting, 44.2% (126/278) considered the department’s atmosphere (friendly, pleasant and collegial) as the most important consideration, 35.4% (101/278) considered professional challenge, 27% (77/278) considered the availability of management roles, 19.4% (56/278) and 19% (54/278) considered flexible work hours and high wages, respectively, and 12.6% (36/278) considered proximity to home. The attitude of the instructor during clinical placement was noted by 3.1% (27/278) as an important consideration when choosing their work setting (note: some participants chose more than one answer).

### Perception of nurses’ tasks

The students’ perceptions of nurses’ roles and tasks in the community vs. the hospitals settings are shown in Table 3. The respondents perceived nursing tasks in the hospital as more important than those of nurses in community care and perceived that clinical placements at the hospital contributed more to students than clinical placements in community care. The respondents also perceived that they have greater knowledge of hospital nurses’ tasks compared to their knowledge on community nurses’ tasks. On average, the respondents’ perceptions regarding nurses’ tasks in each setting did not change during their studies.

**Table 3:** Students’ perceptions of nurses’ roles

Variable	Hospital nurse	Community nurse	***p*** value*
Perceived nurse’s role	3.98 ± 0.48	2.38 ± 0.59	< 0.01
Contribution of clinical placement to the student	4.20 ± 0.65	2.96 ± 0.57	< 0.01
Sense of knowledge as to what the nurse really does	4.41 ± 0.71	3.78 ± 0.93	< 0.01
The extent to which the student’s opinion has changed regarding the nurse’s tasks/functions between starting the nursing program and the last year of studies	3.17 ± 1.03	3.29 ± 1.20	NS

Values are mean±standard deviation on a scale of 1-5; *P value by paired *t* test; NS = not significant

### Perceived characteristics required of nurses

Table 4 summarizes the perceived differences regarding the characteristics required of hospital nurses compared to community nurses. A higher percentage of respondents stated that community nurses, compared to hospital nurses, require abilities for autonomous and individual work, and for exerting their own judgment (89.7% vs. 58.1%) and patience (51.3% vs. 41.9%). A higher percentage of respondents stated that hospital nurses, compared to community nurses, require an ability for team work and acceptance of authority (69.8% vs. 56.4%) as well as good communication capabilities with family members (86.1% vs. 76.9%).

**Table 4:** Students’ perceptions of the characteristics required of hospital nurses vs. community nurses

Characteristics	Hospital nurses	Community nurses
**Number of respondents**	**43**	**39**
Autonomous and individual work, using her own judgment	25 (58.1%)	35 (89.7%)
Patience	18 (41.9%)	20 (51.3%)
Team work and acceptance of authority	30 (69.8%)	22 (56.4%)
Good communication capabilities with family members	37 (86.1%)	30 (76.9%)

### Perceived nurses’ tasks in community care

The respondents perceived that nurses’ tasks in community care include managing chronic patients (diagnosis and treatment, instructing families, coordinating among professionals), regular nursing tasks (electrocardiograms, house visits, taking blood), and to a lesser extent, dealing with uncommon emergency situations.

A significant positive correlation was found between the respondents’ experience during clinical placement and their perceptions of nurses’ tasks, i.e., respondents who had a positive experience in community nursing perceived more positively community nurses’ tasks (*r* = 0.369, *p* < 0.01) while respondents who had a positive experience while working in a hospital perceived the hospital nurses’ tasks more positively (*r* = 0.602, *p* < 0.01).

A univariate regression analysis for predicting the perceived task of community nurses showed a correlation between exposure to community nurses’ tasks and perceiving the tasks of the nurse more positively (*r* = 0.245, *F*(1,278) = 17.6, *β* = 0.155, *p* < 0.01).

A univariate analysis showed that the perception of nurses’ tasks in the hospital/community, the contributions of clinical placement in the hospital/community, and the attitude of the clinical instructor and staff towards the students during clinical placements were statistically significant predictors for the respondents’ positions regarding their preferred work setting after graduation.

The final model of the multivariate linear regression with stepwise selection, to determine contributing factors to community nursing perception, included four independent predictors that significantly accounted for 40.9% of the observed variance between respondents who prefer hospital nursing and those who prefer community nursing (Table 5). Clinical placement in community nursing was the most significant contributor for predicting the difference between respondents who prefer hospital nursing and those who prefer community nursing, explaining 36% of the variance.

**Table 5:** Stepwise regression analysis for predicting community nursing perception

		***B***	SE. B	Beta	***t***	***p*** value
**Step 1**	Clinical placement in community nursing	0.541	.043	0.60	12.46	0.00
**Step 2**	Clinical placement in community nursing	0.485	.044	0.538	10.95	0.00
	Clinical placement in the hospital	− 0.219	0.049	− 0.215	− 4.49	0.00
	Sense of knowledge about hospital nursing	0.33	0.032	0.052	1.04	0.299
**Step 3**	Clinical placement in community nursing	0.473	0.045	0.524	10.55	0.00
	Clinical placement in the hospital	− 0.220	0.049	− 0.216	− 4.48	0.00
	Chronic patient care	0.105	0.049	0.104	2.152	0.032

## Discussion

This study was aimed at understanding the factors that affect fourth (final) year nursing students’ choice of work setting after graduation. Our findings demonstrate that most students retrospectively report beginning their nursing program with an inclination for working in hospitals, and only a few of them showed an interest in community care. The proportion of respondents claiming to be interested in hospital nursing increased at the time of the survey. Similar findings were reported in studies performed in other countries. van Iersel et al. have reported that 71.2% of first-year students from 6 universities across the Netherlands prefer working in general hospitals and only 5.4% prefer community care [20]. Kloster et al. reported that only 7% of Norwegian students chose to practice nursing in the community at the end of their nursing program [21].

Among the respondents who preferred the hospital setting after graduation, there was a clear preference for departments whose contents are perceived by the students as being close to medicine (surgery, emergency medicine, pediatrics). This finding corroborates a previous study conducted among nursing students in Israel [22], and similar findings were also noted in other countries [21, 23]. Interestingly, one third of the students who indicated that they would like to work in a hospital did not specify a preferred department. In another study conducted in Israel, the decision to work in a hospital was considered by nursing students as more important than choosing a specific clinical field. Community nursing was perceived as more monotonous, and the students believed that they would attain more professional experience in a hospital [24]. The belief that hospital experience is essential for developing confidence in nursing skills and clinical judgments was also observed among Australian nursing students [25]. Together, these findings suggest that students perceive the hospital as one setting and view community nursing and hospital nursing as separate worlds [9].

Haron et al. reported that while most nurses (75%) in Israel work 21 to 40 h per week, a greater percentage of community nurses, compared to hospital nurses, work less than 20 h per week (12% vs. 4%). In contrast, a greater percentage of hospital nurses, compared to community nurses, work over 40 h per week (20% vs. 13%) [14]. Although wages of community nurses working in Israel are lower than those of hospital nurses, and have been noted as a barrier to professional development as well as for attracting young people to working in this setting [1], our study showed that only 19% of respondents considered flexible work hours or high wages a contributing factor when choosing a work setting, while the department’s atmosphere, professional challenges, and professional advancement contributed more to the decision on work setting of graduating students.

The finding that the proportion of students who preferred the hospital setting increased between the first and final years of the nursing program, while the number of students who chose community care did not change, suggests that the nursing programs’ curricula and/or the students’ experience during their studies do not convince the students that community care is a “worthy” career choice. This idea is supported by the fact that in the last two decades, the number of hours devoted to community nursing in Israel’s core nursing curriculum has not changed despite the global changes in nurses’ roles. The theoretical studies that are supposed to impart the necessary knowledge and basic skills focus on aspects related to nurses’ role in the hospital. Only 10% of theoretical studies are devoted to community nursing aspects, and students only have one clinical placement in community nursing which constitutes only 15% of the hours allocated to the students’ clinical placement during their studies.

Inadequate exposure to community nursing does not allow the students to fully understand the various aspects of the role and probably conveys a hidden message that community care has a lesser status compared to hospital nursing. Such inadequate exposure probably contributed to the perceptions of students in this study that hospital nurses’ tasks are more important than those of nurses in community care and that clinical nursing training at the hospital contributes more professionally than training in community care. Insufficient exposure to community nursing may also explain why nursing students in our study perceived that they have greater knowledge on hospital nurses’ tasks compared to their knowledge on community nurses’ tasks. Indeed, in a previous study on the views of Israeli leaders and stakeholders in this field, the prevailing opinion was that the exposure to community nursing does not reflect actual work in this setting [19]. Insufficient exposure to community nursing in nursing teaching programs and the absence of appropriate placement opportunities in the community have also been reported for other countries such as New Zealand and Sweden [26, 27]. Canadian nursing students were unaware of the services provided by community nurses, the types of patients, and the community nurse’s areas of practice [28]. Bloomfield et al. [29] reported that nursing students showed greater intention for working in primary healthcare after graduation if they had a placement in this setting. In addition, longer clinical placements have been shown to change students’ perception of community nurses’ roles [30].

The present study shows that students who had a positive experience in clinical community nursing during the study regarded community nursing more positively. This was reflected in the multilinear regression analysis which showed that the contribution of clinical placement in community nursing was the greatest contributor for predicting the difference between respondents who prefer hospital nursing and those who prefer community nursing. This is in line with White’s study [31], which reported that when students choose a job they rely on their predetermined expectations and images about nursing, unless their personal experience during nursing studies suggested otherwise. High-quality placements during nursing studies, which welcomed and supported students were shown to influence nursing career choices [32–35]. On the other hand, settings that did not encourage belongingness, absence of support or supervision, and a poor relationship with the supervisor were associated with reduced learning, increased anxiety and stress, and greater disinclination for nursing programs [36–39].

The current nursing training core curriculum in Israel also does not adequately reflect changes related to the elderly population, especially in the geriatric and mental health fields. In the current curriculum, there is no obligation for clinical placement in geriatrics, and the hours allocated to theoretical learning in this field were decreased. The number of hours of study in the mental health sections of the clinical curriculum has remained unchanged over the past two decades. Given that one out of every five people is likely to develop a psychiatric disorder during his lifetime [9], there is an apparent gap between the existing program and actual practice. Indeed, we found that only a few students claimed that they would like to work in geriatric nursing (1.34%). In a study that examined Israeli nursing students’ attitudes in relation to geriatrics as a future field of practice, only 12% of students considered working in this field while over 60% were not at all interested, and 27% would consider geriatrics only after appropriate training. Intentions to engage in geriatrics were associated with expanding nurses’ authorities as a clinical specialist, positive attitudes toward the elderly, and prior experience in geriatric care. Training in an academic nursing program was found to be a negative predictor [13].

Among the students who preferred working in the community, most said that they would like to work with younger people. This is similar to the results of Haron et al. who found that over 60% of students at the end of their studies will not consider working in geriatrics as a field of occupation [40]. The field of psychiatry also received an exceptionally low priority for future student engagement, at 3.3%. Other studies have also shown that nursing students show preferences for working with healthy people or with those who are more likely to recuperate, rather than working with psychiatric patients, the elderly or with chronically ill patients [22, 33, 41–45].

Despite the finding that students’ preferred work setting does not change during their nursing program, some studies have reported that students’ preferences can be changed [21, 46]. White [31] reported that 88% of students believed that their career choice was affected by their clinical placement. Nursing schools that exposed students to community nursing showed a change in students’ preferences. For example, the addition of community studies that included health promotion and preventive medicine courses, teamwork, and ongoing clinical experience in various areas of the community led students to reevaluate the community nurse’s role, to understand the work environment and eventually to want to remain in this work setting [47].

### Policy recommendations

To encourage nursing students to choose community nursing, students’ exposure to community work should be increased by integrating community-focused content into the training program in order to show that community nursing is an integral part of the overall approach to patient care and expectations of nurses. Bouchaud and Gurenlian suggested that aspects of community nursing should be incorporated into each course of the nursing curriculum so that community and hospital nursing would be taught alongside rather than separately [48]. The Institute of Medicine has recommended providing nurses with tools to plan, implement, and evaluate strategies for prevention, protection, and preservation of patients’ health in their natural environment within the community [49], and the Association of Community Health Nursing Educators (ACHNE) has recommended including comprehensive clinical training related to community nursing in nursing curricula [50–52]. The curriculum should also emphasize holistic aspects of prevention and health promotion, chronic care in the community, and end of life [53].

Additionally, clinical placement in the community should be expanded to expose students to the variety of community nursing roles, and the challenges involved in its different aspects and specific patient populations including, the elderly, mental health patients, and high-risk communities. Students’ experience during clinical placement contributes to shaping the student’s opinion of the nurse’s role within that field [54]. Positive experiences in community nursing may lead the student to choose this area as a future field of practice, but if students perceive their experience as negative they are unlikely to want to work in this setting [4, 26, 55]; therefore, an emphasis should be placed on providing high-quality placements within the community and supportive supervisors.

Lastly, nursing leaders, particularly those in nursing training programs, should ensure that community nursing is perceived as a positive and interesting career option. This may be done by strengthening the identity of community nurses and including community nurses as teachers and role models in these programs.

### Strengths and limitations

The strength of this study is the large sample of students from all nursing education frameworks across Israel; therefore, our findings may be generalized to the entire nursing student population in Israel. The proportion of male students in our respondent population (23.4%) was more than double the proportion of men in the Israeli nurse population (10.5%) [14]. According to Ashkenazi et al. the low status of the nursing profession deters Israeli men from choosing nursing as a career. Additionally, men tend to choose nursing due to financial constraints significantly more frequently than women [56]; Haron et al. reported that 82% of Israeli male nurses work in hospitals in comparison to 70% of female nurses. In comparison, 8% of male nurses work in community settings in comparison to 21% of female nurses [14]. The proportion of Arab nurses in our study was also higher at 25.6% than that reported for the general nurse population in Israel (12%). Due to the low percentage of respondents who preferred the community setting, we could not further analyze statistically the differences between community and hospital preferences in terms of gender and ethnicity.

Although the researcher explained to the students that the questionnaires are completed anonymously, because the questionnaires were completed in the classroom, the students’ answers may have been affected by social desirability bias. Students’ answers about their preferred post-graduate work setting when they started their nursing program may have been affected by a recall bias; however our findings regarding the proportion of students who prefer hospital nursing upon entering nursing programs are in line with findings of other studies.

## Conclusions

The results of our study reflect the students’ lack of interest in choosing community nursing after graduation and their perception of community nursing as a low status field. The students’ inclination to work in hospitals does not change during the course of their studies. The students’ perceptions regarding community nurses’ roles are based upon limited information, which does not allow the student a deep sense of understanding of the role and does not reflect the community nurses’ real work. On the other hand, when students feel that they do have deep knowledge and understanding of “what community nurses do” and the confusion and ambiguity are small, community nurses’ roles are perceived more positively.

The conflict between students’ preferences for working in hospitals, and the growing needs of community nursing will only be solved by planning long-term strategies to increase the attractiveness of community nursing. Such strategies would have an influence on nursing students’ views regarding their perception of community nursing and its challenges. To change this historical trend, collaborations are required between educational frameworks and community health organizations. Meaningful and valuable training in the community will help broaden students' perspectives and encourage them to consider work in this field.

## Data Availability

The datasets used and/or analyzed during the current study are available from the corresponding author on reasonable request.
